# Supplementary material for: Fibroblast Activation Protein-Targeting Minibody-IRDye700DX for Ablation of the Cancer-Associated Fibroblast with Photodynamic Therapy
Source: Cells. 2023 May 18;12(10):1420. doi: 10.3390/cells12101420 (PMC10217124; doi:10.3390/cells12101420)
Supplement: Supplementary file 1 [file cells-12-01420-s001.zip › cells-2329614-supplementary.pdf]

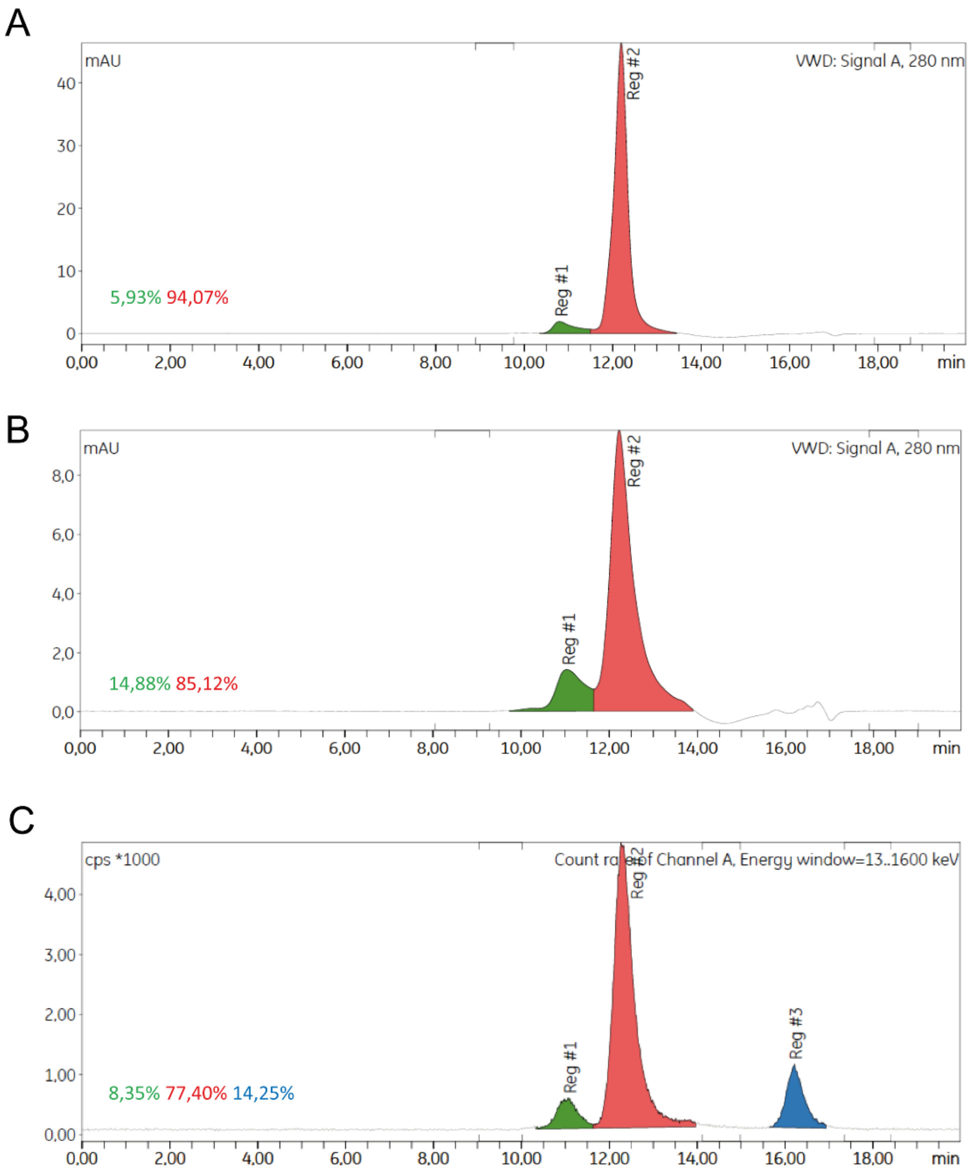

Supplementary figure S1 FPLC showing the A280 channel of A) the native minibody and B) the conjugate DTPA-MB-700DX, and the radioactive channel of C) ^{111}In-labelled DTPA-MB-700DX. The colored regions are manually annotated and percentages are quantified based on area under the curve. Green indicates the region of probable aggregated conjugates, red indicates the region of the minibody monomer en blue indicates free ^{111}In.

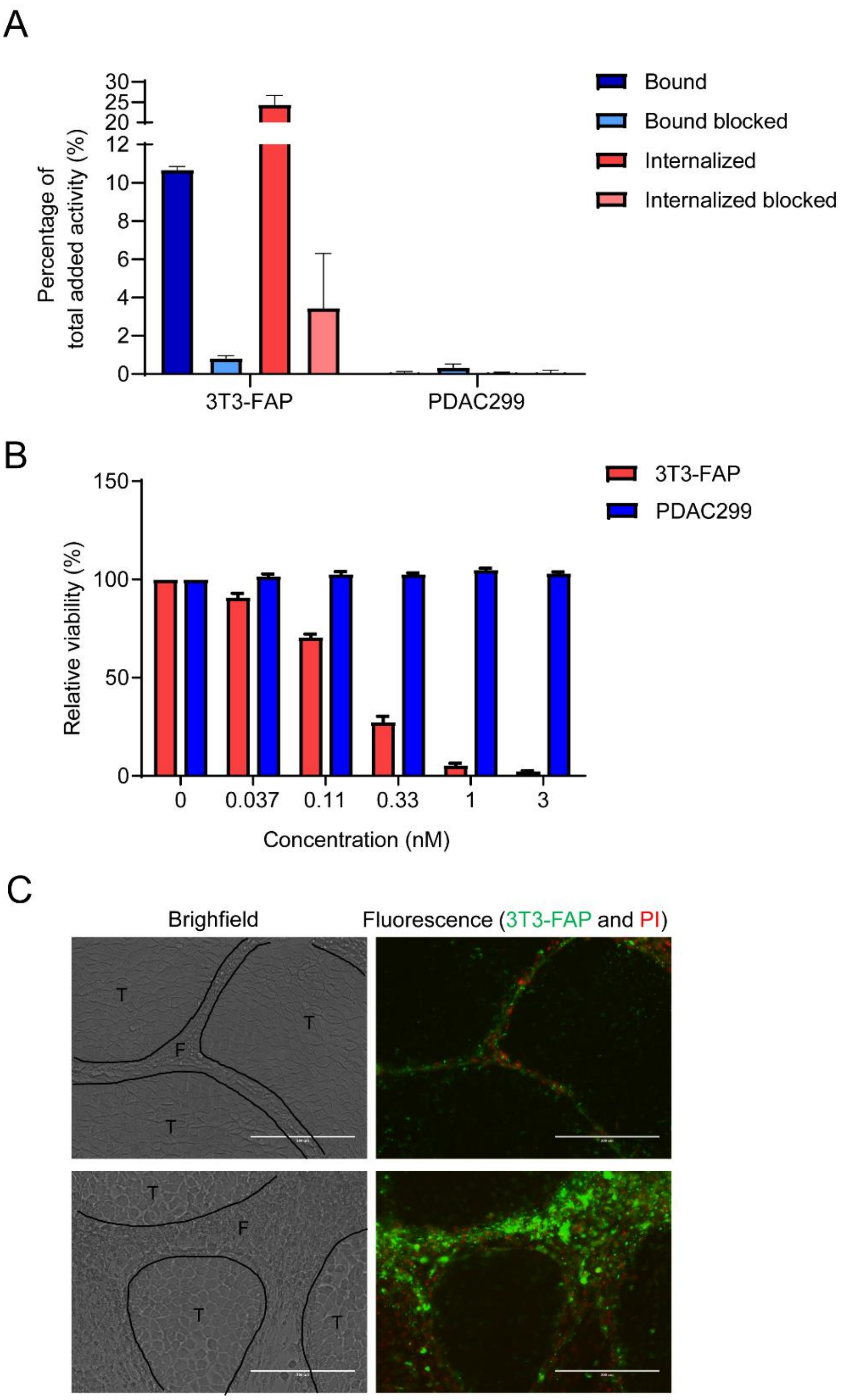

Supplementary Figure S2 Lack of association of DTPA-700DX-MB with PDAC299 cells in vitro as shown in A) a binding assay illustrating bound and internalized fractions of ^{111}In-labelled DTPA-700DX-MB after 2 hours incubation of 3T3-FAP or PDAC299 cells at 37 °C B) a PDT assay illustrating cell viability of 3T3-FAP and PDAC299 cells after incubation of varying

doses of DTPA-700DX-MB for 2 hours and subsequent irradiation with 60 J/cm^2 200 mW/cm^2 690 nm light. C) Cocultures of PDAC299 cells (T in brighfield image) and 3T3-FAP cells (F in brightfield image and green in fluorescent image) were incubated with 3 nM DTPA-700DX-MB and irradiated with 60 J/cm^2 690 nm light. After treatment, dead cells were visualized through incubation with propidium iodide (red nuclei in fluorescence image). Note that the red nuclei are located primarily in the regions with 3T3-FAP cells.

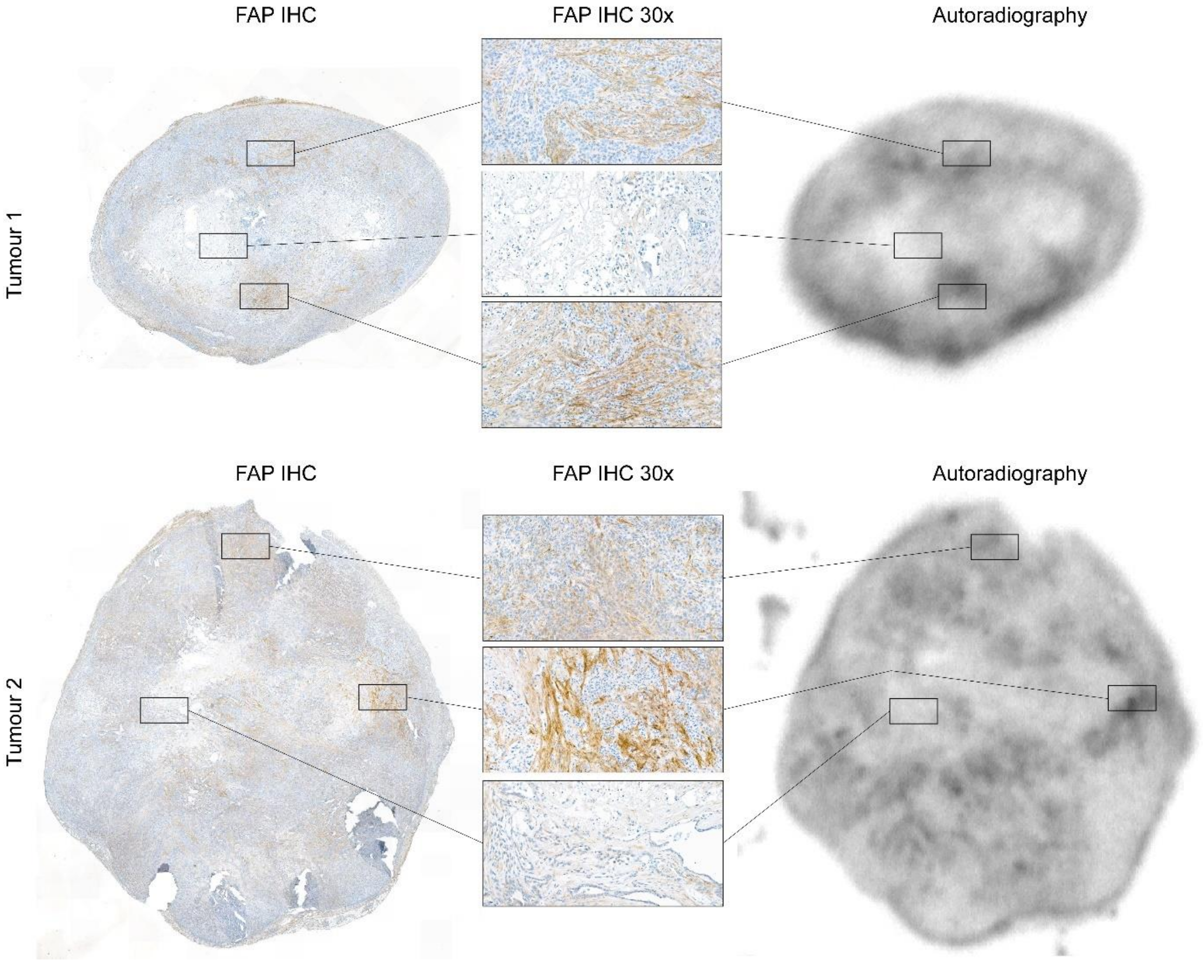

Supplementary Figure S3 Autoradiography of subcutaneous PDAC299 tumour sections of two mice upon injection of 0.3 nmol 10 MBq ^{111}In-labelled DTPA-700DX-MB and correlation with FAP expression as visualized by anti-FAP immunohistochemistry in high and low expressing regions.

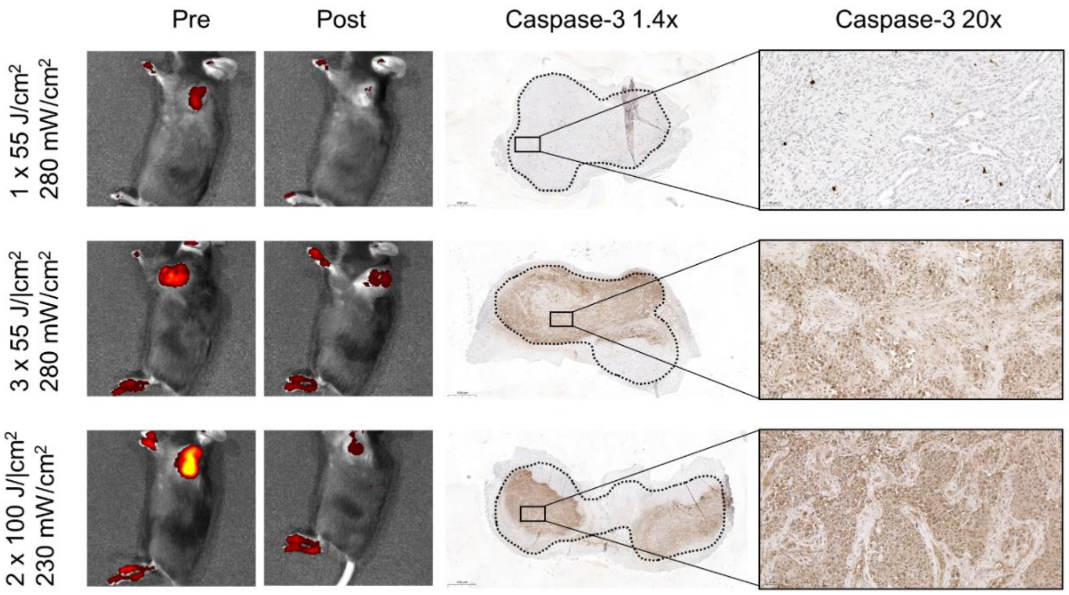

Supplementary Figure S4 In vivo efficacy of targeted photodynamic therapy with DTPA-700DX-MB. Mice carrying subcutaneous PDAC299 tumours were injected with 0.6 nmol DTPA-700DX-MB. 24 hours after injection fluorescence was visualized (pre), then tumours were exposed to the indicated light dose (rate) of 690 nm light and fluorescence was visualized again (post). Tumours were formalin fixed and paraffin embedded and induction of apoptosis was assessed with IHC.

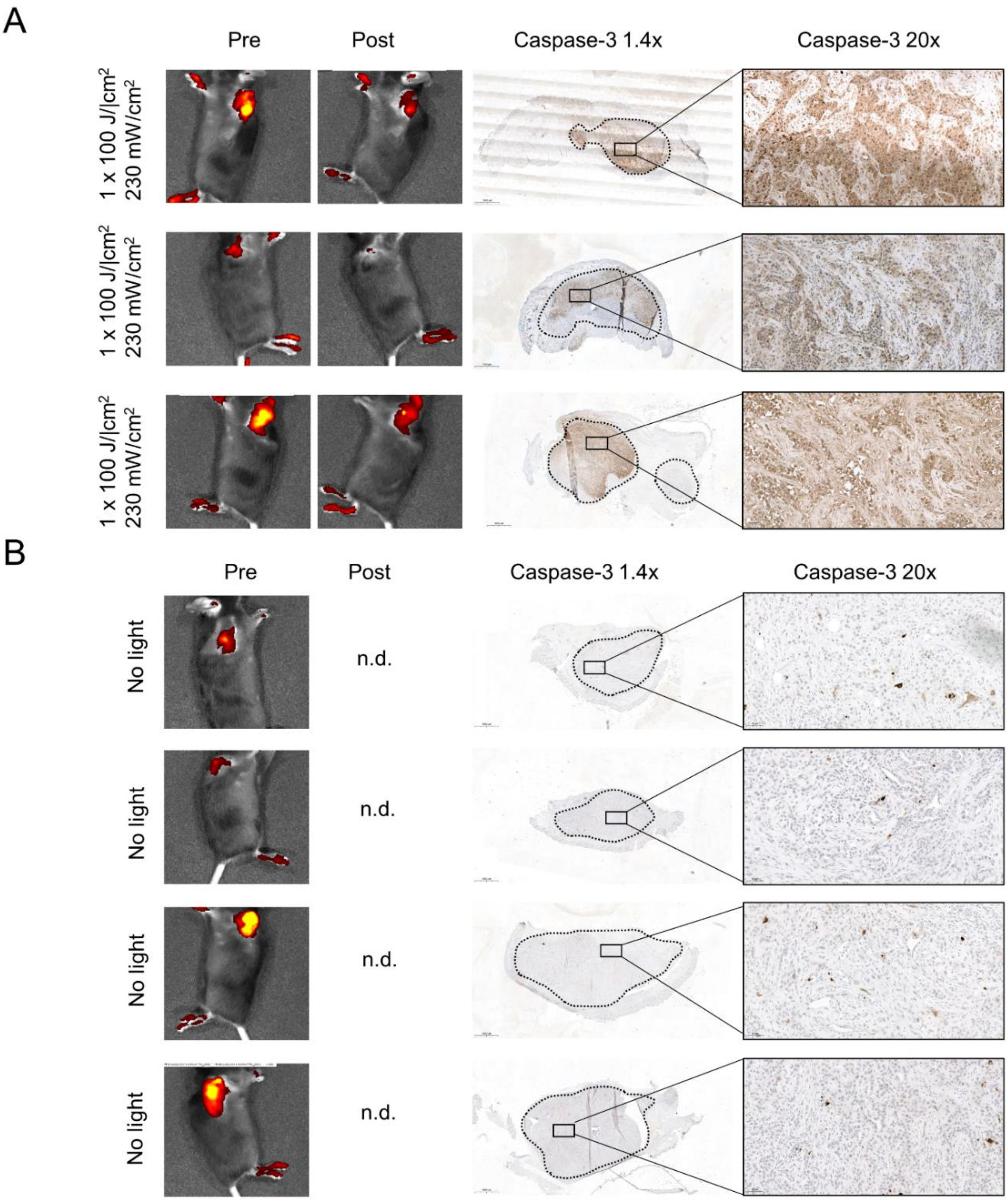

Supplementary Figure S5 A) In vivo efficacy of targeted photodynamic therapy with DTPA-700DX-MB. Mice carrying subcutaneous PDAC299 tumours were injected with 0.6 nmol DTPA-700DX-MB. 24 hours after injection fluorescence was visualized (pre), then tumours were exposed to the indicated light dose (rate) of 690 nm light and fluorescence was visualized again (post). Tumours were formalin fixed and paraffin embedded and induction of apoptosis

was assessed with IHC. B) Contralateral tumours that were not exposed to light were formalin fixed and paraffin embedded, and induction of apoptosis was assessed with IHC.

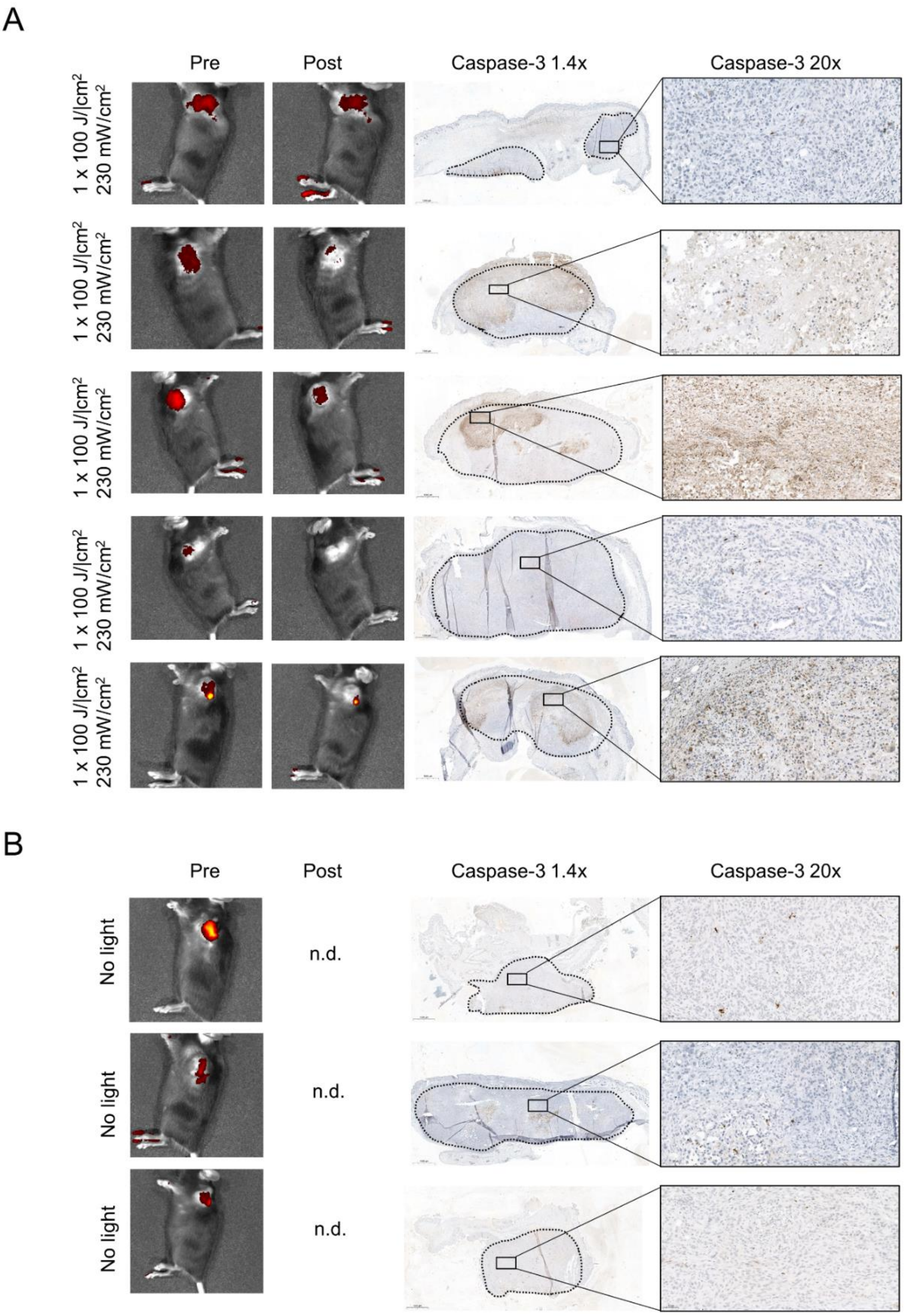

Supplementary Figure S6 A) Control group for FAP-tPDT in vivo. Mice carrying subcutaneous PDAC299 tumours were injected with PBS. 24 hours after injection (auto)fluorescence was visualized (pre), then tumours were exposed to the indicated light dose (rate) of 690 nm light. Tumours were formalin fixed and paraffin embedded and induction of

apoptosis was assessed with IHC. B) Contralateral tumours that were not exposed to light were formalin fixed and paraffin embedded, and induction of apoptosis was assessed with IHC.

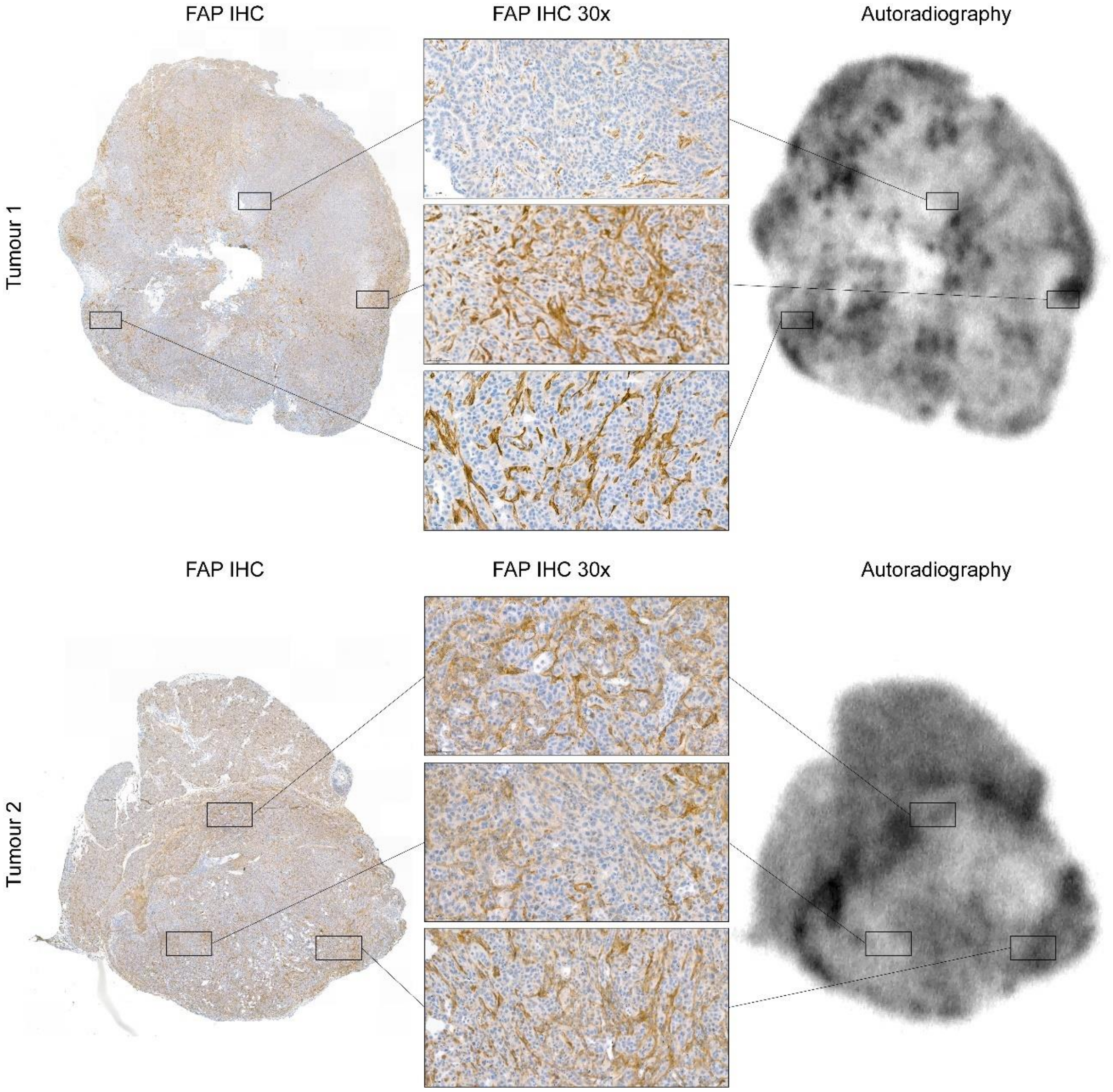

Supplementary Figure S7 Autoradiography of orthotopic PDAC299 tumour sections of two mice upon injection of 0.3 nmol 10 MBq ^{111}In-labelled DTPA-700DX-MB and correlation with FAP expression as visualized by anti-FAP immunohistochemistry in high and low expressing regions.

	4 hours (N=5)	24 hours (N=7)	24 hours blocked (N=3)	48 hours (N=5)
**Blood**	7.5 ± 0.47	0.88 ± 0.12	1.03 ± 0.09	0.39 ± 0.04
**Muscle**	0.80 ± 0.19	0.73 ± 0.14	0.74 ± 0.16	0.62 ± 0.13
**Tumour**	6.60 ± 0.89	7.29 ± 0.94	4.36 ± 1.45	5.31 ± 0.32
**Lung**	4.62 ± 0.29	1.44 ± 0.29	1.90 ± 0.16	0.94 ± 0.07
**Liver**	34.78 ± 2.24	27.50 ± 2.69	24.71 ± 4.56	21.64 ± 1.63
**Spleen**	7.61 ± 0.62	6.31 ± 0.76	6.42 ± 0.91	4.53 ± 0.54
**Pancreas**	1.75 ± 0.21	1.58 ± 0.23	1.43 ± 0.21	1.00 ± 0.05
**Stomach**	2.05 ± 0.06	1.30 ± 0.16	1.61 ± 0.38	0.90 ± 0.03
**Kidney**	11.00 ± 0.62	7.32 ± 0.62	11.98 ± 2.56	5.02 ± 0.53
**Tibia + bone marrow**	9.85 ± 1.09	6.81 ± 0.50	3.64 ± 0.80	5.63 ± 0.56

Supplementary Table S1 Uptake in various tissues calculated as percentage of the injected activity dose per gram of tissue (%IA/g).

	5 x 10^3 cells (N=3)	5 x 10^3 cells blocked (N=2)	2,5 x 10^4 cells (N=4)
**Blood**	1.35 ± 0.17	1.05 ± 0.12	1.21 ± 0.36
**Muscle**	0.78 ± 0.17	0.74 ± 0.18	0.78 ± 0.09
**Tumour**	8.97 ± 2.01	7.89 ± 2.03	9.28 ± 1.44
**Lung**	1.25 ± 0.26	1.77 ± 0.36	1.73 ± 0.29
**Liver**	28.37 ± 2.20	27.36 ± 4.23	26.75 ± 2.41
**Spleen**	7.90 ± 0.62	8.04 ± 1.31	8.15 ± 1.48
**Pancreas**	5.91 ± 2.20	5.40 ± 0.93	4.71 ± 2.90
**Stomach**	1.66 ± 0.29	1.66 ± 0.08	2.12 ± 0.34
**Kidney**	19.50 ± 0.84	25.57 ± 2.30	21.16 ± 1.00
**Tibia + bone marrow**	6.61 ± 0.55	4.15 ± 0.12	6.96 ± 0.74

Supplementary Table S2 Uptake in various tissues calculated as percentage of the injected activity dose per gram of tissue (%IA/g).
